# Curcumin: Useful add-on for Rheumatic Diseases?

**DOI:** 10.3390/jcm11102908

**Published:** 2022-05-20

**Authors:** Stylianos Tomaras, Gernot Keyßer, Eugen Feist

**Affiliations:** 1 Department of Rheumatology, HELIOS Clinic Vogelsang-Gommern, 39245 Vogelsang-Gommern, Germany; eugen.feist@helios-gesundheit.de; 2 Clinic for Internal Medicine II, Department of Internal Medicine, University Hospital Halle (Saale), Martin Luther University Halle-Wittenberg, 06120 Halle, Germany; gernot.keyszer@uk-halle.de

**Keywords:** curcumin, turmeric, rheumatology, anti-inflammatory, bioavailability

## Abstract

Plant-derived nutraceuticals are proposed as new key instruments to represent a profound “back to basics” shift in medical treatment. Data accumulated over the past ten years suggest that curcumin, the major active compound of the turmeric plant, has anti-inflammatory properties. It has yet to be determined whether the anti-inflammatory profile of curcumin is potent enough to justify the application of this substance as a nutritional supplement for patients with rheumatic diseases. To address this question, the most relevant in vitro studies that investigate the mechanism of action of curcumin were reviewed in this article. In addition, a total of 18 animal and human trials were evaluated. The pleiotropic, anti-inflammatory and immunomodulatory effects of curcumin were observed in animal studies. In addition, human trials demonstrated promising findings. In these studies, curcumin was able to reduce the expression of proinflammatory cytokines, lower the level of the C-reactive protein and improve clinical parameters. A limiting factor of the application of curcumin is the inconsistent bioavailability of the substance. Therefore, new formulations have been developed to improve the pharmacodynamic profile of curcumin. The future acceptance of the substance is dependent on new controlled clinical trials with a standardised formulation of curcumin administered as well as standard of care.

## 1. Introduction

Even in an era of biologic disease-modifying antirheumatic drugs (bDMARDs) and targeted synthetic disease-modifying antirheumatic drugs (tsDMARDS), only a minority of patients with rheumatic diseases reach full remission of their disease. Due to a high proportion of primary and secondary non-responders, switching between therapies is frequent. Even among rheumatoid arthritis (RA) patients with a Disease Activity Score in 28 joints (DAS28) C-reactive protein (CRP) remission, a proportion of 74–77% does not achieve “true” Boolean remission. The latter is defined by a number of tender joints ≤1, a number of swollen joints ≤1, a CRP level ≤1 mg/dL, and a patient’s global assessment (0–10 cm visual analogue scale (VAS) score) ≤1 [1]. Furthermore, according to patient-reported outcomes (PROs), the feeling of pain does not necessarily correlate with inflammatory markers. Significantly, the pain intensity does not always correlate with the measured disease activity in RA, especially when secondary osteoarthritis is present. Therefore, approximately 30% of responders still experience significant pain [2]. This also accounts for patients with psoriatic arthritis (PsA) and axial spondyloarthritis (axSpA), where enthesitis-related pain may not respond well to DMARD treatment [3]. As a consequence, analgesics and steroids are still widely used in rheumatology with numerous side effects.

New insights into the pathomechanisms of rheumatic diseases highlight the role of the environment and the microbiome. The statement attributed to Hippocrates, “Let food be thy medicine and medicine be thy food”. (These exact words were never found in the *Corpus Hippocraticus*, meaning they are probably a commonly used misquotation. However, Hippocrates spent time studying the relationship between nutrition and disease onset and did (inarguably) say: “All disease begin in the gut”, without being able to even imagine in 370 BC the impact of the gut microbiome on modern medicine) should be once again brought to the fore.

Turmeric, the botanical name of which is *Curcuma longa*, is an aromatic herbaceous plant found in Southeast Asia that belongs to the ginger family. India is the world’s largest producer, consumer and exporter of turmeric. The bright orange powder derived from the turmeric rhizome has been used for thousands of years in Ayurvedic cuisine and in traditional holistic medicine as a healing herb. Curcumin, the most bioactive component of turmeric, has attracted much attention over the past two decades. Scientific interest in curcumin was triggered by epidemiological studies showing lower rates of colorectal and prostate cancer [4], and Alzheimer’s disease [5] in India compared to Western countries. Currently, 70 ongoing clinical trials, mainly in oncology, obesity-related metabolic diseases and neurology, are being conducted to investigate curcumin’s health-promoting activities (search on 22 February 2022) [6]. Additionally, promising results indicating the positive effect of curcumin on Crohn’s disease and ulcerative colitis were outlined in the 2019 guidelines of the British Society of Gastroenterology on the management of inflammatory bowel disease in adults [7] as well as in the updated 2019 S3-guidelines on ulcerative colitis of the German Society for Digestive and Metabolic Diseases [8].

Two of the first animal model studies that demonstrated that curcumin alleviates inflammation in arthritis were published in 2003 [9] and 2006 [10]. Over time, an extensive body of literature has developed on this topic. Moreover, it has been suggested that curcumin could be used as a preventive supplement-therapy for patients with periodontitis and concomitant rheumatic diseases [11]. Significantly, novel technologies could facilitate the transition from a nutritional supplement to an anti-rheumatic drug.

## 2. Methods

A PubMed search was conducted from January 2002 to February 2022, using the following medical subject headings (MeSH), either alone or in combination with the appropriate Boolean operators: curcumin, turmeric, arthritis, musculoskeletal pain, analgesics, anti-inflammatory agents, autoimmune disease* and rheumatic disease*. This strategy yielded more than 380 results. The authors selected the year 2002 as a cut-off for the selection of articles regarding in vitro and animal models, whereby the oldest citation dates back to 2007. As for human studies, a focus was set on publications not older than 10 years. Studies of complex combinations of dietary supplements were excluded, but studies reporting negative results were not. For this informative review, we focused on 18 publications (Table 1).

## 3. Results

### 3.1. In Vitro Studies

**Figure 1:**
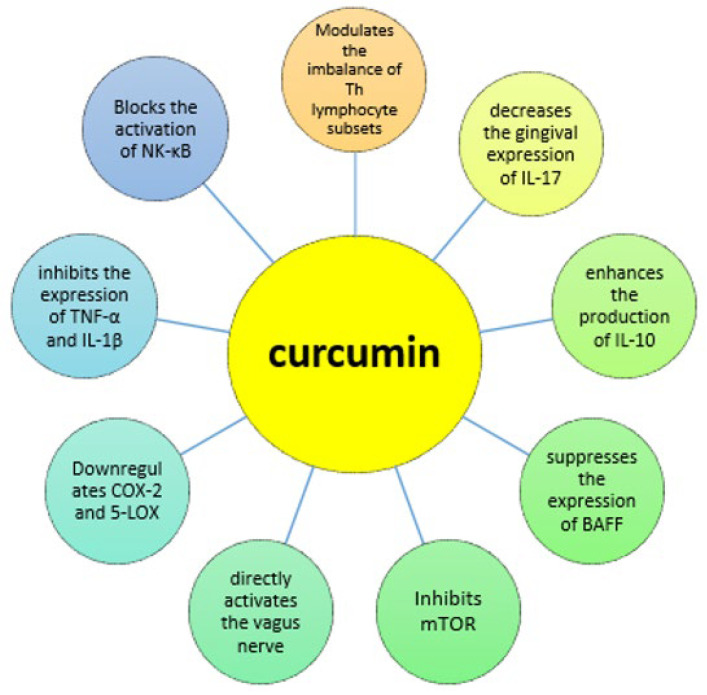
Mechanism of action. COX-2 = cyclo-oxygenase-2, 5-LOX = 5-lipoxygenase, mTOR = mammalian target of rapamycin, BAFF = B cell activation factor, IL = interleukin, Th = T helper cells, NK-κB = nuclear factor kappa-light-chain-enhancer of activated B cells, TNF = tumor necrosis factor.

The pleiotropic anti-inflammatory and immunomodulatory effects of curcumin are listed in Figure 1. It could have a potential role in manipulating immune responses through the modulation of different T helper subsets [30]. It blocks the activation of nuclear factor kappa-light-chain-enhancer of activated B cells (NK-κB) and inhibits the expression of tumor necrosis factor-α (TNF-α) and interleukin-1β (IL-1β) [31,32]. In addition, it inhibits arachidonic acid-derived pro-inflammatory mediators by downregulating COX-2 and 5-lipoxygenase (5-LOX) [33]. Curcumin is also able to inhibit the Janus Kinase 2/Signal Transducers and Activators of Transcription (JAK2/STAT) pathways in different types of myeloproliferative neoplasms [34], a finding that is remarkable given the growing relevance of JAK/STAT-inhibition in the treatment of rheumatic diseases. Furthermore, curcumin can enhance the production of IL-10, a potent anti-inflammatory cytokine [35].

### 3.2. Animal Studies

Curcumin could directly activate the vagus nerve, thereby attenuating inflammatory processes. This hypothesis is supported by animal models, in which rats with collagen-induced arthritis (CIA) who underwent vagotomy did not respond to curcumin [36]. Vagus nerve stimulation is a novel therapeutic approach with promising research results that were presented at the 2019 American College of Rheumatology (ACR) annual meeting [37]. Moreover, curcumins pharmacological effect depends on intestinal interactions, possibly through an increase in somatostatin secretion [38].

Another animal study reported that curcumin inhibits the mammalian target of the rapamycin (mTOR) signalling pathway, which is involved in the pathogenesis of synovitis in RA [12], a condition induced by fibroblast-like synoviocytes [39]. In the study, rats were divided into the following four groups: control animals (*n* = 10), CIA without treatment (*n* = 8), CIA treated with curcumin (*n* = 8) and CIA treated with rapamycin (*n* = 8). Rapamycin (syn. sirolimus) is an inhibitor of the mTOR signalling pathway. Curcumin significantly decreased swelling (measured by the hind-paw oedema volume), inhibited the mTOR pathway (detected through immunohistochemical analysis of the synovium) and alleviated the expression of the pro-inflammatory cytokines IL-1β and TNF-α and the matrix metalloproteinases MMP-1 and MMP-3 in the serum and synovium. Histopathological assessments of the paw joints also revealed reduced synovial hyperplasia in the curcumin group compared to the controls. These results were very similar to those of the CIA treated with rapamycin group.

Furthermore, curcumin could suppress the interferon-ɣ (IFN-ɣ)-induced expression of B cell activation factor (BAFF) and thereby attenuate the progression of CIA in mice [40]. In addition to its anti-arthritic properties, curcumin was able to modulate experimental *Porphyromonas gingivalis*-induced periodontitis in rats through a decrease in the gingival expression of IL-17. This finding is interesting as *P. gingivalis* induces the citrullination of peptides and is suggested to contribute to the development of RA [41].

Another study demonstrated the therapeutic effects of intravenous (iv.) native curcumin (*n* = 6) in rats with adjuvant-induced arthritis (AIA), and tested curcumin-loaded nanoemulsions via oral administration (*n* = 6) vs. intravenous methotrexate (MTX) (*n* = 6) and placebo (*n* = 6). The duration of this trial was 14 days. Nanoemulsions can protect hydrophobic curcumin from rapid intestinal degradation by incorporating it into oil droplets. Compared with the placebo group, all three treatment groups achieved a decrease in the levels of TNF-α and IL-1β (*p* < 0.001) in both the synovial fluid and serum and a decrease in paw swelling (*p* < 0.001), the histopathologic synovitis score (*p* < 0.001) and NK-κB expression (*p* < 0.001). The anti-arthritic effects of curcumin were similar to those of MTX. A parallel study of curcumin bioavailability indicated that curcumin-loaded nanoemulsions exhibit a three-fold greater absorption than curcumin suspension.

A further study investigated histological changes in rats with CIA after 28 days of treatment with either orally administered curcumin (*n* = 6) or betamethasone (*n* = 6). Both substances were dissolved in olive oil. The control group consisted of six rats without CIA and animals with CIA that received olive oil only. Curcumin reduced cell infiltration and cartilage/bone erosion significantly, but not synovial hyperplasia or pannus formation. In contrast, betamethasone treatment had significant effects on all parameters tested [14]. The effects of combination treatment with curcumin and steroids were analysed in a controlled study of rats with AIA. The samples received curcumin and/or prednisolone either as monotherapy or in various combinations. The results suggest an increased efficacy of prednisolone in inhibiting inflammatory cytokines when given in combination with curcumin [15].

A study group compared the anti-inflammatory actions of curcumin-loaded solid lipid nanoparticles (SLNs) to those of naproxen in rats with AIA over a period of 14 days. SLNs efficiently delivered curcumin across the gastrointestinal mucosa and protected against its enzymatic degradation through the gut and liver. Both curcumin SLNs and naproxen significantly improved pain, as measured by thermal and mechanical hyperalgesia as well as mechanical allodynia. Both substances improved mobility and joint stiffness and reduced paw volume and serum TNF-α and CRP levels (with curcumin showing a stronger effect on CRP than naproxen). The effects of curcumin SLNs were also equivalent to those of naproxen with respect to the reduction in the radiological scores. Notably, curcumin reduced serum anti-cyclic citrullinated peptide (CCP) antibody levels, whereas naproxen did not [16]. In a similar study, curcumin exhibited anti-inflammatory effects comparable to those of indomethacin in mice with CIA; both attenuated synovitis and bone erosion. A histological analysis of the paws on day 38 after the first immunization with collagen showed that orally administered curcumin suppressed MMP-1, MMP-3 and TNF-α-stimulated fibroblast-like synoviocytes in a dose-dependent manner [17]. Another research team concluded that curcumin administered every other day for two weeks in mice with CIA effectively suppressed the inflammatory response by inhibiting TNF-α, IL-1β, MMPs and the prostaglandins E_2_ and COX-2 [18].

Curcumin micelles are spherical aggregates with hydrophilic surface molecules that incorporate hydrophobic curcumin powder in their centre. The combination of curcumin with boswellic acids in a micellar delivery system performed better than native curcumin in reducing the paw volume in rats with AIA. Both compounds reduced TNF-α, IL-6 and CRP levels and parameters of oxidative stress synergistically [19].

### 3.3. Human Studies—RA

A randomized, double-blind, placebo-controlled trial demonstrated a significant improvement in the signs and symptoms of RA with the use of a novel formulation of highly bioavailable curcumin. Curcuminoids, turmeric essential oil and water-soluble fractions of turmeric were built into a matrix, thereby protecting hydrophobic curcumin. This could be achieved by polar–nonpolar sandwich technology. The bioavailability of curcumin in this product increased 10-fold compared to that of the native extract. Patients were divided into three groups and received low-dose matrix curcumin (250 mg twice a day, *n* = 12), high-dose matrix curcumin (500 mg twice a day, *n* = 12) or placebo (*n* = 12). All patients completed 90 days of treatment without reporting any serious adverse events. Biochemical parameters, such as creatinine, bilirubin, alkaline phosphatase and cholesterol levels, did not change during the trial. The DAS28 values in both treatment arms decreased from 4.51 ± 0.64 to 2.14 ± 0.16 (*p* < 0.001) in the 250 mg matrix curcumin group and from 5.29 ± 0.54 to 1.80 ± 0.36 (*p* < 0.001) in the 500 mg matrix curcumin group. In the placebo group, the DAS28 value did not change. CRP levels dropped significantly (< 0.001) in both verum arms, whereas in the placebo arm the CRP level did not change at all. Furthermore, the total number of swollen joints was reduced in both treatment groups. Significant decreases (*p* < 0.001) in the erythrocyte sedimentation rate (ESR), rheumatoid factor (RF), number of tender joints and ACR20 response were also observed. A major limitation of this study is the lack of information regarding concomitant medications—particularly prednisolone—during the trial [20]. Moreover, these results are often not achieved by much larger studies with highly effective biologics. Additionally, the cohort described with 2/3 male patients in a female-dominated disease, the discrepancy between an incredibly high blood sedimentation rate and the comparatively low CRP is highly surprising. Such a rapid and massive response of the rheumatoid factor has not been described even with B cell-directed therapies and cannot be explained.

The safety and effectiveness of curcumin in patients with RA were investigated in a randomized, single-blinded pilot trial. Overall, forty-five patients were divided into three groups and received 500 mg capsules of native curcumin twice a day (*n* = 15), 50 mg diclofenac capsules twice a day (*n* = 15) or combination therapy (*n* = 15). The duration of this trial was eight weeks, with DAS28 response as the primary endpoint. All three treatment regimens reduced the total number of swollen joints from 12.15 to 0.36 (*p* < 0.05) in the curcumin group, from 16.6 to 1.83 (*p* < 0.05) in the diclofenac group and from 11.5 to 0.42 (*p* < 0.05) in the combination group. Curcumin monotherapy also achieved a 52% change in CRP levels from 5.34 to 2.56 mg/dL (*p* < 0.05), whereas diclofenac alone or in combination with curcumin failed to do so. The DAS28 in all three arms significantly improved and decreased from 6.4 to 3.5 (*p* < 0.05) in the curcumin group, from 6.4 to 3.5 (*p* < 0.05) in the diclofenac group and from 6.7 to 3.8 (*p* < 0.05) in the combined therapy group. The highest response rate was achieved in the curcumin monotherapy group, as demonstrated by the ACR20, ACR50 und ACR70 scores of 93%, 73% and 33%, respectively. Regarding safety, adverse events were reported more frequently in the diclofenac treatment arm than in the curcumin treatment arm. However, it is also not clear how many patients were on concomitant steroids during the trial [21]. Moreover, the results suggest that curcumin as a monotherapy has the potential to reduce the number of swollen joints from 12 to <1 within 8 weeks. The question is whether these surprising results can be generalised to a broader population. There is a case for not acknowledging all possible bias, at least in the discussion section of this publication. Such high clinical remission rates are uncommon in the field of rheumatology.

Finally, in 2019, a randomized, double-blind, controlled trial with 65 patients with RA was published. Curcumin nanomicelle, when administrated three times a day for twelve weeks as an add-on therapy to the patients’ medication, did not improve DAS28 significantly vs. placebo [22]. There were no significant changes in the laboratory parameters either.

### 3.4. Human Studies—OA

A randomized, single-blinded, controlled, multicentre trial on the efficacy of curcumin in patients with knee osteoarthritis (OA) had two arms: 185 patients were assigned to a native curcumin group and treated with 1500 mg/day of curcumin, and 182 patients received 1200 mg/day of ibuprofen. The duration of the study was four weeks. The primary endpoint was the Western Ontario and McMaster Universities (osteo) arthritis index (WOMAC) score, a tool that assesses pain, stiffness and physical function in osteoarthritis. Both treatments led to significant (*p* < 0.001) improvements in the WOMAC global, WOMAC pain, WOMAC stiffness and WOMAC joint function-scores, with no between-group differences. In addition, the number of reported adverse events was similar in both arms [23].

In a smaller study, 19 patients with knee OA assigned to the curcumin group showed significantly improved VAS for pain and WOMAC global scores compared with twenty patients that received placebo. No relevant adverse events were reported in this randomized, double-blind, placebo-controlled trial over six weeks [24]. Another study group tested the combination of curcumin and diclofenac in patients with knee OA in a randomized controlled trial. Patients were administered 75 mg/day diclofenac with either placebo (*n* = 44) or 1000 mg curcumin (*n* = 44). There were no significant between-group differences in VAS pain score or joint function after three months of treatment [25]. One randomized, non-inferiority trial compared the efficacy and safety of 500mg turmeric capsules twice daily vs. 650mg paracetamol three times daily for 6 weeks. The results suggest curcumin is non-inferior in reducing pain and found it to be safe and more effective than paracetamol in reducing CRP and TNF-α [26]. Another randomized, double-blind, placebo-controlled study with 101 participants showed greater improvements in pain and functional assessment in the curcumin group. On top of that, painkillers were reduced in 37% compared to 13% on placebo [27].

A systematic meta-analysis of seven randomized trials to search for evidence of the treatment of knee OA with curcumin included a total of 797 patients. Studies involving combinations of curcumin with other herbal supplements were excluded, as were studies with a duration of less than 4 weeks. The average daily dose of curcumin varied from 180 mg to 2 g, which was administered orally. In the majority of trials, curcumin was applied in its native form. Overall, the risk of bias, as assessed by the Cochrane criteria, was moderate. Curcumin significantly reduced knee pain, achieving a reduction in the VAS score of—3.45 (*p* < 0.001) and improved quality of life compared to placebo. However, the improvement in stiffness and physical function of the knee joint, as measured by the WOMAC score, was not as prominent compared to the effects of ibuprofen. No serious adverse events were reported [28]. A meta-analysis analysed five randomized studies with a total of 599 patients with all forms of osteoarthritis. The authors concluded that curcumin could significantly reduce the VAS pain score and improve the WOMAC score, without any safety signals [29]. However, a major limitation of both meta-analyses is the diversity in the methodologies used in the primary studies. Since these studies administered curcumin in several different formulations and at various doses, a comparison of the bioavailability between studies was not possible.

### 3.5. Poor Bioavailability Is a Major Issue

Although curcumin has been referred to as “Indian solid gold” [42], due to its numerous therapeutic properties, its unstable pharmacologic properties, namely its unreliable absorption, limit its clinical usage. This uncertainty has cast doubt on its role in medicine and has impaired its pharmaceutical development. The fast and extensive metabolism of curcumin in the liver [43] with its low absorption by the intestinal wall lead to reduced bioaccessibility with very low blood concentrations, even upon the oral intake of curcumin at doses as high as 10 or 12 g [44]. In a study in 2006, 24 healthy volunteers were asked to take a single dose of curcumin ranging from 0.5 to 12 g. Curcumin was detected in the sera of only two participants after doses of 10 and 12 g of curcumin were taken. When consumed in doses below 8 g, curcumin was not detectable [45]. The pharmacokinetic profile of curcumin was also evaluated in a study of 36 patients with Alzheimer’s disease who received a placebo (*n* = 12), 2 g/d curcumin (*n* = 12) or 4 g/d curcumin (*n* = 12). Maximum plasma concentrations remained in the low nanomolar range after 24 weeks of administration [46]. Several other studies were performed to quantify the blood levels of curcumin in patients with different types of neoplasms [47], chemotherapy-refractory colorectal cancer [48] and pancreatic cancer [49] and in smokers with pre-malignant lesions for colorectal cancer [50]; all of these studies displayed very inconsistent results highlighting the poor pharmacodynamic reliability of curcumin.

### 3.6. New Curcumin-Formulations to Circumvent Its Poor Bioavailability

Given the beneficial properties of curcumin, research groups around the globe have sought novel strategies to counteract its poor absorption and the extent of its metabolic elimination. Some of these formulations, such as nanoparticles and micelles, have been used successfully in the food and cosmetics industries for years.

The concomitant application of the adjuvant piperine, the natural alkaloid of black pepper, exhibited a 20-fold increase in the bioavailability of curcumin, compared with its native form, based on the area under the plasma concentration–time curve (AUC) [51]. Turmeric essential oils as adjuvants also enhanced the oral bioavailability of curcumin 7-fold [52]. Phospholipid–phytochemical complexes with lecithin increased the bioavailability of curcumin 4-fold [53]. Furthermore, micronized curcumin displayed a 9-fold increase in AUC [54]. Curcumin in hydrophilic nanoparticles showed a 27-fold increase in AUC as a result of enhanced solubility and a subsequent improvement of gastrointestinal absorption [55]. Significantly, a newly developed micellar delivery system enhanced the bioavailability of curcumin 185-fold based on AUC [54]. In this study, the Cmax of curcumin following the administration of liquid curcuminoid micelles (containing 410 mg curcumin) at a dose of 500 mg exceeded that of the oral administration of 8 g of native curcumin.

## 4. Discussion and Conclusions

Currently, the market of nutraceuticals is developing rapidly. In 2016, the global curcumin business was worth half a billion US dollars, with an expected growth rate of 13% by 2025 [56]. Our paper reviews the latest research articles about the beneficial properties of curcumin as an anti-inflammatory agent in rheumatology. At the same time, it highlights a significant discrepancy: despite the fact that the number of preclinical in vitro and animal studies of curcumin has increased rapidly (approx. 380 hits in Medline), there are only three published randomized clinical trials in humans. The main reason for this is the poor bioavailability of curcumin. Recent developments in nanoparticle and liquid micelle technology will probably solve this problem in the near future and stimulate the design of new interventional trials in humans. A limitation of our work is the fact that the selection of articles followed the personal impression of the authors regarding their quality and relevance.

Although most preclinical studies have indicated that curcumin has an immunomodulatory effect, it is likely that curcumin will find its place as a complementary therapy and not as a DMARD. It is premature to assume that curcumin could be used in the treat to target (T2T) concept for patients with rheumatic diseases [57]. Future research should investigate the possible steroid- and NSAID-sparing effects of curcumin. Another area of future research could be the impact of curcumin on cardiovascular comorbidities in patients with rheumatic diseases. Curcumin has already shown promising signals in patients with metabolic syndrome [58]. The same accounts for further studies to assess the impact of curcumin on the inflammation in gout, since in vitro studies have shown positive signals [59].

Novel anti-inflammatory therapies are often tested in patients with RA first, since it is the most common and well-examined disease. However, patients with PsA and axSpa could benefit from curcumin as well. This group of patients often present with metabolic and cardiovascular comorbidities, similar to RA patients [60]. In addition, curcumin may act as an anti-inflammatory agent in several skin disorders, including psoriasis [61].

Overall, curcumin has been associated with an acceptable and consistent safety profile, apart from mild side effects such as nausea and diarrhea. It would be interesting to see in future studies whether the hitherto excellent tolerability could be compromised by these new resorption-promoting modifications with prolonged use, such as micelles. If the expected effects cause a relevant and sustained downregulation of TNF, interleukins and other pro-inflammatory cytokines, undesirable risks may be incurred.

To conclude, new advanced curcumin formulations are of interest for investigation in controlled trials in rheumatic diseases. The outcome of such trials should not only focus on disease activity of the underlying condition, but also on metabolic and cardiovascular endpoints as well as on the sparing effect of NSAIDs and steroids.

## Figures and Tables

**Table 1:** Overview of the 15 animal and human trials evaluated in this paper.

Reference	Disease	Number of Subjects/Patients	Study Duration	Curcumin Formulation	Outcomes
Dai Q. et al. (2018) [12]	CIA	34 rats	21 days	Native curcumin	Paw swelling ↓, IL-1β and TNF-α ↓, proteinases MMP-1 and MMP-3 in the serum and synovium ↓, histopathologic synovial hyperplasia ↓
Zheng Z. et al. (2015) [13]	AIA	24 rats	14 days	Native curcumin and curcumin-loaded nanoemulsions	TNF-α and IL-1β ↓, paw swelling ↓, histopathologic synovitis score ↓
Kamarudin TA. et al. (2012) [14]	CIA	24 rats	28 days	Native curcumin	cell infiltration and cartilage/bone erosion ↓, no changes in synovial hyperplasia and pannus formation.
Kuncha M. et al. (2014) [15]	AIA	60 rats	21 days	Native curcumin	anti-arthritic effect of prednisolone ↑ in the curcumin group
Arora R. et al. (2015) [16]	AIA	58 rats	14 days	Curcumin-loaded solid lipid nanoparticles (SLNs)	Both curcumin SLNs and naproxen: pain ↓, mobility ↑, joint stiffness ↓, paw volume ↓, serum TNF-α and CRP levels ↓, radiological scores ↓. Noticeably, curcumin reduced serum anti-CCP-antibody levels, whereas naproxen did not.
Mun SH. et al. (2009) [17]	CIA	30 mice	15 days	Native curcumin	synovitis ↓ and bone erosion ↓, MMP-1 and MMP-3 ↓
Moon DO. et al. (2010) [18]	CIA	NK.	14 days	Native curcumin	TNF-α ↓, IL-1β ↓, MMPs ↓, prostaglandins E_2_ and COX-2 ↓
Khayyal MT. et al. (2018) [19]	AIA	NK.	21 days	Combination of curcumin with boswellic acids in a micellar delivery system	paw volume ↓, TNF-α ↓, IL-6 and CRP levels ↓
Amalraj A. et al. (2017) [20]	RA	36 patients	90 days	Curcuminoids, turmeric essential oil and water-soluble fractions of turmeric built into a matrix, therefore protecting hydrophobic curcumin, via polar-nonpolar sandwich technology	DAS28 ↓, CRP and ESR ↓, RF ↓, number of swollen joints and tender joints ↓, ACR20 response ↑
Chandran B. and Goel A. (2012) [21]	RA	45 patients	8 weeks	Native curcumin	number of swollen joints ↓, CRP levels ↓, DAS28 ↓, ACR20, ACR50 und ACR70 responses ↑
Javadi M. et al. (2019) [22]	RA	65 patients	12 weeks	Curcumin nanomicelles	Positive changes in DAS28, tender and swollen joints, but not significant
Kuptniratsaikul V et al. (2014) [23]	Knee OA	367 patients	4 weeks	Native curcumin	improvement in the WOMAC global, WOMAC pain, WOMAC stiffness and WOMAC joint function scores
Panahi Y. et al. (2014) [24]	Knee OA	39 patients	6 weeks	Native curcumin	improved VAS-pain and WOMAC-global scores
Pinsornsak P. and Niempooq S. (2012) [25]	Knee OA	88 patients	3 months	Native curcumin	There were no significant differences in VAS pain score or joint function between the groups.
Singhal S. et al. (2021) [26]	Knee OA	144 patients	6 weeks	Native curcumin	Curcumin as effective as paracetamol
Lopresti AL. et al. (2021) [27]	Knee OA	101 patients	8 weeks	Native curcumin	Pain ↓, knee functional tests ↑, concomitant use of painkillers ↓
Onakpoya IJ. et al. (2017) [28]	Knee OA	797 patients	Meta-analysis	Various formulations	knee pain ↓, quality of life ↑
Wu J. et al. (2019) [29]	All types of OA	599 patients	Meta-analysis	Various formulations	VAS pain score ↓, WOMAC score ↑

CIA = collagen-induced arthritis, mTOR = mammalian target of rapamycin, IL = interleukin, TNF-α = tumour necrosis factor-α, MMP = matrix metalloproteinase, AIA = adjuvant-induced arthritis, NK-κB = nuclear factor kappa-light-chain-enhancer of activated B cells, MTX = methotrexate, CRP = c-reactive protein, CCP = cyclic citrullinated peptide, NK = not known, COX-2 = cyclo-oxygenase-2, RA = rheumatoid arthritis, DAS28 = Disease Activity Score in 28 joints, ESR = erythrocyte sedimentation rate, RF = rheumatoid factor, ACR = American College of Rheumatology, OA = osteoarthritis, WOMAC = Western Ontario and McMaster Universities (osteo) arthritis index, VAS = visual analogue scale, Up arrow = increased, Down arrow = reduced.

## Data Availability

Not applicable.
